# Evaluation of urine sample for diagnosis of visceral leishmaniasis using rK-39 immunochromatographic test in Northwest Ethiopia

**DOI:** 10.1371/journal.pone.0263696

**Published:** 2022-02-07

**Authors:** Tahir Eyayu, Melashu Yasin, Lemma Workineh, Tegenaw Tiruneh, Henok Andualem, Meslo Sema, Shewaneh Damtie, Aynework Abebaw, Birhanu Getie, Desalegn Andargie, Barnabas Achaw, Wubet Taklual

**Affiliations:** 1 Department of Medical Laboratory Sciences, College of Health Sciences, Debre Tabor University, Debre Tabor, Ethiopia; 2 University of Gondar Compressive Specialized Hospital Laboratory, University of Gondar, Gondar, Ethiopia; 3 Department of Public Health, College of Health Sciences, Debre Tabor University, Debre Tabor, Ethiopia; Bangladesh Agricultural University, BANGLADESH

## Abstract

**Background:**

Visceral leishmaniasis is the most severe form of leishmaniasis which ranks second in mortality and fourth in morbidity. Parasitological diagnostic techniques with splenic aspirate remain the gold standard. However, sample collection is risky, painful, and difficult. Alternatively, serological techniques provide good diagnostic accuracy using serum sample that is difficult for applying on small children and in the field. So, finding alternative non-invasive and self-collected samples like urine is very important. Thus, the study aimed to evaluate the diagnostic performance of the rK-39 strip test using urine for diagnosis of visceral leishmaniasis.

**Methods:**

A multicenter institutional-based cross-sectional study was conducted from November 2019 to March 2021 at Northwest Ethiopia. Sociodemographic information was collected using a structured questionnaire. Blood sample and midstream urine sample were collected for rK-39 test. Data were entered into Epi-data version 4.2 and analyzed using SPSS version 24.0. Diagnostic performance parameters of urine-based rK-39 rapid test, i.e. sensitivity, specificity, positive and negative predictive values, positive and negative likelihood ratios (LR+/−), and diagnostic accuracy were determined on contingency table by using serum-based rK-39 test result as a reference. An agreement between urine and serum-based rK-39 test was statistically determined by kappa value.

**Result:**

In total, 300 subjects, age ranged between 7 and 60 years, were included in the study. The overall sensitivity, specificity, positive predictive value, negative predictive value and diagnostic accuracy of urine-based rK-39 test were found to be 98.0% (95% CI: 93.0% - 99.8%), 95.5% (95% CI: 91.6% - 97.9%), 91.6% (95% CI: 85.2%– 95.4%), 98.9 (95% CI: 96.0%– 99.7%), and 96.33% (95% CI: 93.53–98.16%), respectively. Additionally, there was a strong agreement between the results obtained on rK-39 ICT using urine and serum samples (kappa = 0.92; P < 0.001).

**Conclusion:**

Urine-based rK-39 ICT had an excellent high sensitivity, specificity and strong agreement with serum-based rK-39 ICT results. This indicates that urine sample would be a promising noninvasive and easy to collect sample for diagnosis of VL in field and rural settings.

## Background

Visceral leishmaniasis (VL), also known as kala-azar, is the most severe form of leishmaniasis which is caused by the obligate intracellular protozoan parasites *Leishmania donovani* complex [1]. It is one of the world’s most neglected tropical diseases which ranks the second in mortality and fourth in morbidity [2]. The global burden of VL is estimated at 0.2 to 0.4 million cases, resulting in estimated 50,000 deaths every year. Over 95% of cases can be fatal without early diagnosis and proper treatment and results in 2.357 million disability-adjusted life years lost, representing a significant rank among communicable diseases [3, 4].

In recent years, it is estimated that 50,000 to 90,000 new cases of VL occur worldwide annually, with only between 25 to 45% of the cases were reported to WHO. About 90% of the global burden of VL is found only in seven countries, four of which are in Eastern Africa (Sudan, South Sudan, Ethiopia and Kenya), and the remaining three are India, Bangladesh and Brazil [5, 6].

Eastern Africa is the second highest burdened region after the Indian subcontinent [3]. Ethiopia is one of the endemic country in this area with the highest number of VL cases next to Sudan [7]. The national incidence estimated based on self-reported cases is up to 4500 new cases per annum and over 3.2 million people are at risk of VL infection. Furthermore, around 33% of the landmass in northwestern, northeastern, western and southeastern parts of the country is highly suitable for the transmission of VL [8, 9]. In Ethiopia, from 8 regions (Tigray, Amhara, Oromia, Somali, Afar, Gambella, Benshangul Gumuz and Southern Nations and Nationalities People’s Regional State) VL confirmed cases have been reported [10].

Parasitological diagnostic techniques with splenic aspirate remain the gold standard for the diagnosis of VL. Alternative standard means of diagnosis in most countries are lymph node or bone marrow aspirates. The sensitivity of such diagnostic methods is highly variable and depends on the sampling procedure and technical skills of the physician or personnel performing the tests. However, these procedures are painful, potentially dangerous, require considerable skills, and thus are not practical at the primary hospital and health center levels. Also, very few practitioners currently have the skills to perform these invasive aspirates and very limited numbers of these biopsies are taken [1, 11].

Molecular techniques are very excellent in sensitivity and diagnostic accuracy in laboratory-based methods for diagnosis of clinical VL through amplifying parasite DNA. These methods are not currently able to be adapted in developing countries due to the expensive infrastructure and technical expertise required. The overall cost associated with a PCR assay per sample is two times greater than rK39 Immune Chromatographic Test (ICT) [1, 12]. Therefore, these are far from being suitable diagnostic methods for developing countries.

In VL endemic areas, accurate, sensitive, specific and rapid types tests are important for proper treatment and control of the disease. Serological techniques provide a good diagnostic accuracy as long as they are used in combination with a standardized clinical case definition. A rapid ICT based on a recombinant 39-amino acid repeat antigen (rK39) test is the most used tool in the diagnosis of the VL [2, 13, 14]. However, this technique should be performed using a serum that needs blood collection and centrifugation. The process is difficult for applying in the field conditions and resented by some groups of patients such as small children [15, 16]. To overcome the existing shortcomings of serum-based diagnostic methods, finding alternative non-invasive and self-collected samples like urine is very important.

Leishmania nucleic acid as free circulating DNA can cross the glomerular filtration barrier and be found in urine. According to various reports, kidney impairment occurs during the acute phase of VL infections and leishmania and /or leishmanial particles and antibodies have been detected in the patient’s urine. Therefore, urine seems to be a promising biological sample for the diagnosis of VL, since its collection is simple and doesn’t need invasive procedures thus more convenient for the patient [17–19].

There are some studies in different parts of VL endemic areas that describe the best diagnostic performance of rK39 strip tests using urine samples [13, 17, 20–22]. However, studies regarding the test performance of rK-39 test by using urine are not conducted in our country, even though the prevalence is high. Therefore, this study was aimed to assess the diagnostic performance of the rK-39 test by using urine samples in VL endemic areas of Ethiopia.

## Methods and materials

### Study area

The study was implemented at five health centers, one hospital and one town in the South Gondar Zone. South Gondar Zone is found in Amhara regional state located 664 km from the capital city Addis Ababa. South Gondar Zone has 1 comprehensive specialized hospital, 9 district hospitals, 97 health centers, and 394 health posts. According to the Ethiopia population projection of 2017, an estimated total population of South Gondar Zone is 2,364,603 of whom were 1,168,285 females and 1,196,318 were males [23].

The health facilities were selected for the study purpose based on the endemicity of VL. Based on this, we have selected Debre Tabor town from a non-endemic area for VL free healthy controls. Whereas from VL endemic area, four health centers (Addis zemen, Yifag, Shehoch Tahara, and Ambo Meda Health center) and Addis zemen Primary hospital at Libo kemkem district and Wereta health center from Fogera district were selected. The endemic districts are located in a black cotton clay soil flat plain (1800–2000 meters above sea level). Most of the area is flooded during the rainy season (July–September) and dried up during the dry season (November–March), resulting in deep cracks in the soil surface, which could turn into breeding sites for the vector of the pathogen [24].

### Study design and period

A multicenter institutional-based cross-sectional study was carried out from November 2019 to March 2021 at selected Hospitals and health centers in the South Gondar Zone, Northwest Ethiopia.

### Sample size determination

The sample size of study population was determined with regard to the following formula used for diagnostic studies

$$n=\frac{{\left(Z_{\frac{\alpha }{2}}\right)}^{2}p(1-p)}{d^{2}}$$

where *p* is pre-determined value of sensitivity (or specificity) that is ascertained by previous published data [25]. Assuming that the sensitivity of the rK-39 test by using urine in previous study was 95% [20] and considering a 95% confidence interval (CI) and 5% of margin of error, the sample size needed was at least 73 VL patients. For controls, at least 97 individuals were required, assuming 93.3% specificity [20] and considering a 95% CI with a 5% of margin of error. To increase the accuracy of the study, the number of study participants has to be increased. Thus, a total of 300 study participants (100 VL patients and 200 controls) were enrolled in the study.

### Study subjects

An overall of 300 individuals were included in this study. In total, 250 subjects were enrolled from different health facilities which are found in endemic areas for VL and 50 from the non-endemic district (Debre tabor town) of South Gondar Zone. The subjects enrolled were distributed into four groups:

#### Group I

100 VL patients from endemic areas, who were positive with the serum-based rK-39 strip test and had a combination of more than two of the following symptoms or signs, fever for at least two weeks, hepatomegaly and/or splenomegaly, cytopenia were enrolled in this study.

#### Group II

Healthy controls from endemic areas- consisted of 100 individuals considered healthy by their assessment and/or clinical exam, living in an endemic area for VL, who stayed asymptomatic for the last six months and were negative by the serum-based rK-39 strip test.

#### Group III

Healthy controls from non-endemic areas- consisted of 50 individuals considered healthy by their assessment and/or clinical exam, living in the non-endemic area for VL, who stayed asymptomatic for the last six months and were negative by the serum-based rK-39 strip test. These individuals were from Debre Tabor town.

#### Group IV

Other infectious disease controls- consisted of 50 patients with a confirmed diagnosis of malaria (N = 20), tuberculosis (N = 15), and typhoid fever (N = 15) and who were negative by the serum-based rK-39 strip test.

However, for all the above groups, individuals with a confirmed diagnosis of HIV, pregnant and breastfeeding females, individuals who have a history of VL and were treated with anti-leishmanial drugs were excluded from this study. On the other hand, for group I–III other diseases with the possibility of cross-reactivities, like cutaneous leishmaniasis, tuberculosis, malaria, and typhoid fever were excluded from the study.

### Data collection procedure

#### Questionnaire survey

Socio-demographic characteristics of study participants were collected using a structured questionnaire written in Amharic (the local language). Clinical information and patient history were assessed by trained clinical nurses. A structured data collection form was used to record the results of different laboratory test results.

#### Sample collection and laboratory analysis

*Blood sample collection and serum separation*. Three ml of venous blood was collected by following standard operating procedures. The blood sample was dispensed into serum separator test tubes. Blood was allowed to clot by leaving it undisturbed at room temperature for 15–30 minutes after collecting. Clotted sample was centrifuged at 3000 revolutions per minute for 5 minutes to remove the serum [26]. The resulting supernatant is serum, where the rK-39 strip test was performed and interpreted according to the manufacturer’s instructions.

*Urine sample collection*. Individuals were given screw-capped urine vials with their identification number labeled on it. Each participant also had to give a midstream urine sample of about 20 ml. Firstly, a small amount of urine is passed into the toilet and then collecting urine is started into the container. After collecting the sample, passing the urine is finished into the toilet [27]. Fresh urine samples were examined within 15 minutes for VL antibodies. Both blood and urine samples were collected during the same visit. The two samples from a single individual were tested independently by different laboratory personnel.

*Serum rK-39 test*. VL antibodies were determined by using Kalazar *Detect*™ Rapid Test (InBios Inc., USA). The Kalazar *Detect™* Test is a qualitative, membrane-based immunoassay for the detection of VL antibodies in human serum. The membrane is pre-coated with rK39 on the test line region and chicken anti-protein A on the control line region. The test was also performed as per the protocol of the manufacturer instruction. Briefly, 20 μl of serum samples were applied to the base of nitrocellulose strips impregnated with rK-39 antigen. After being air-dried, 2–3 drops of test buffer were added, and the strip was placed upright. The appearance of a lower red band (control) indicated the proper functioning of the test while the appearance of an upper red band indicated the presence of anti-rK-39 antibodies signifying a positive test [20, 28].

*Urine rK-39 test*. was determined by using a similar test strip with serum rK-39 test. But for urine assay, 3 drops of urine sample were applied directly to the strip without adding any test buffer [20].

In both tests, the test was considered positive when both bands (control and test) appeared within 10 minutes and negative when only the upper control band appeared. A faint/weak band was considered positive, whereas only a test line band was considered an invalid test [21].

### Data management and analysis

Data was entered by using Epi-data version 4.2 and transferred to SPSS version 24.0 for statistical analyses. Quantitative data was summarized using proportions and means. With the results from serum based rK-39 test as a reference, sensitivity and specificity of the urine-based rK-39 test were calculated using two-by-two cross tables. Positive predictive value (PPV) and negative predictive value (NPV) are used to assess the clinical relevance of a urine-based rK-39 test. Furthermore, the likelihood ratio of a positive test (LR+) and the likelihood ratio of a negative test (LR–) were determined. Results were considered as good if LR+ >10 and LR–<0.1 which indicates strong evidence for the disease or the absence of disease, respectively [29]. The kappa (κ) coefficient associated with the relative strength of agreement was interpreted as <0.59, 0.6–0.79, 0.8–0.9 and >0.9 considered as weak, moderate, strong and almost perfect respectively [30]. Finally, a *p*-value <0.05 was considered statistically significant.

### Ethics approval and consent to participate

This project received ethical approval from the Research Ethics Committee of the Debre Tabor University, Collage of Health Sciences, on October 1, 2019 (approval number CHS/224/19). Permission was obtained from South Gondar Zone Health Department administrative. Informed verbal and written consent and accent were obtained from each participant. Confidentiality of the information were maintained.

## Results

### Socio-demographic characteristics of study participants

A total of 300 individuals were included in the present study. The majority of the participants were males 188 (62.7%) and 145 (48.3%) was in the age group of 18–34 years. More than half of the study participants (64.3%) reported that they are rural dwellers. Regarding educational status, only eleven percent of the study participants completed grade 12. A majority of study participants were orthodox Christian 245 (81.7%), farmer 93 (31.0%) and single 142 (47.3%) based on their marital status (Table 1). The mean age of VL patients was 28.97±10.92 with an age range between 7 and 57 years and control participants were 31.61±12.89 with an age range between 8 and 60 years.

**Table 1:** Socio-demographic characteristics of the study subjects at South Gondar zone, Northwest Ethiopia.

Characteristics	Categories	Cases, n = 100 (%)	Control, n = 300 (%)	Total, n = 400 (%)
**Sex**	Male	70 (70.0)	118 (59.0)	188 (62.7)
	Female	30 (30.0)	82 (41.0)	112 (37.3)
**Age in years**	< 18	10 (10.0)	30 (15.0)	40 (13.3)
	18–34	56 (56.0)	89 (44.5)	145 (48.3)
	35–44	24 (35.0)	57 (28.5)	81 (27.0)
	≥45	10 (10.0)	24 (12.0)	34 (11.3)
**Residence**	Urban	32 (32.0)	75 (37.5)	107 (35.7)
	Rural	68 (68.0)	125 (62.5)	193 (64.3)
**Religion**	Orthodox	82 (82.0)	163 (81.5)	245 (81.7)
	Muslim	16 (16.0)	34 (17.0)	50 (16.7)
	Protestant	2 (2.0)	3 (1.5)	5 (1.7)
**Occupation**	Farmer	42 (42.0)	51 (25.5)	93 (31.0)
	Merchant	12 (12.0)	22 (11.0)	34 (11.3)
	Government employee	5 (5.0)	22 (11.0)	27 (9.0)
	Student	28 (28.0)	61 (30.5)	89 (29.7)
	House wife	10 (10.0)	27 (13.5)	37 (12.3)
	Others*	3 (3.0)	17 (8.5)	20 (6.7)
**Educational status**	Illiterate	24 (24.0)	36 (18.0)	60 (20.0)
	Only read and write	23 (23.0)	58 (29.0)	81 (27.0)
	Primary school	32 (32.0)	40 (20.0)	72 (24.0)
	Secondary school	17 (17)	33 (16.5)	50 (16.7)
	Above grade 12	4 (4.0)	33 (16.5)	44 (12.3)
**Marital status**	Single	47 (47.0)	95 (47.5)	142 (47.3)
	Married	42 (42.0)	85 (42.5)	127 (42.3)
	Divorced	8 (8.0)	19 (9.5)	27 (9.0)
	Widowed	3 (3.0)	1 (0.5)	4 (1.3)

Note

*- Daily laborer and unemployed

#### Diagnostic performance of rK39 ICT test using urine

A total of 100 individuals were positive for an rK-39 test by using the serum sample. From which 98 have been found to be positive by the urine sample yielding a sensitivity of 98.0% (95% CI: 93.0% - 99.8%) for the detection of VL while false-negative results were found for 2 (2.0%) of 100 VL samples. On the other hand, a total of 200 individuals tested negative for rK-39 test by using the serum sample. Of those samples, which tested negative by serum sample, 191 were negative, while 9 were positive by rK-39 test strip using a urine sample. This translates into a specificity of 95.5% (95% CI: 91.6% - 97.9%) (Table 2).

**Table 2:** Urine rK-39 test results compared to rK-39 test results by using serum sample, Northwest Ethiopia.

		Serum rK-39 test result		
		Positive n (%)	Negative n (%)	Total n (%)
**Urine rK-39 test result**	**Positive n (%)**	98 (98.0)	9 (4.5)	107 (35.7)
	**Negative n (%)**	2 (2.0)	191 (95.5)	193 (64.3)
	**Total n (%)**	100 (33.3)	200 (66.7)	300 (100.0)
**Measures of diagnostic performance**			**Value (95% CI)**	
**Sensitivity**			98.0% (93.0% - 99.8%)	
**Specificity**			95.5% (91.6% - 97.9%)	
**Positive predictive value**			91.6% (85.2%– 95.4%)	
**Negative predictive value**			98.9% (96.0%– 99.7%)	
**Likelihood ratio for positive test**			21.78 (11.49–41.26)	
**Likelihood ratio for negative test**			0.02 (0.01–0.08)	
**Diagnostic Accuracy**			96.33% (93.53%– 98.16%)	

Of those tested positive by the urine based rK-39 test, 98 were positive by the serum based rK-39 test, while nine were negative. This translates into a positive predictive value of 91.6% (95% CI: 85.2%– 95.4%). Of those tested negative by the urine based rK-39 test, 191 were negative according to the serum based rK-39 test while two were positive. This results a negative predictive value of 98.9 (95% CI: 96.0%– 99.7%).

The positive likelihood ratio of the urine-based test for VL was 21.78 (95% CI: 11.49–41.26) while the negative likelihood ratio was at 0.02 (95% CI: 0.01–0.08). Considering all study groups, the rK-39 ICT test achieved the highest diagnostic accuracy (95% CI) using urine in relation to serum, 96.33% (95% CI: 93.53–98.16%) (Table 2).

#### Performance of rK-39 test by using urine among different study groups

Considering all of the control groups together, rK39 test by using urine showed that the specificity of 95.5% with 95% CI of 91.6% - 97.9%. Among non-endemic healthy controls, urine based rK-39 test showed a 100% of specificity, but, in endemic healthy controls the test by using urine sample gave 4 false positive result that interpreted as 96% specificity. Despite the fact that the specificity of urine based rK-39 test were lower in other disease patient groups as compared with other control groups (Table 3).

**Table 3:** Sensitivity and specificity of rK-39 ICT test performed using urine sample from VL patients and non-VL individuals for diagnosis of VL.

Groups	Urine Sample				
	Subjects (n)	Positive (n)	Sensitivity (%)	Specificity (%)	95% CI
VL patients	100	98	98.0	NA	92.7–99.8
All controls	200	9	NA	95.5	91.6–97.9
EHC	100	4	NA	96.0	90.1–98.9
OIDC	50	5	NA	90.0	78.2–96.7
NEHC	50	0	NA	100	92.9–100

**Abbreviations:** NA, not applicable; 95% CI, sensitivity or specificity at 95% confidence interval; EHC, endemic healthy control; OIDC, other infectious disease controls; NEHC, non-endemic healthy control

#### Agreement between rK39 tests in serum and urine sample

Further, rK-39 test by using urine sample showed excellent agreement with rK-39 test by using serum sample (k = 0.92; P < 0.001). Thus, antibodies against the diagnostic antigens like rK-39 antigens of leishmania species were readily and specifically detected in the urine sample of VL patients.

## Discussion

This study was designed to assess the performance of non-invasive samples for the diagnosis of VL patients. According to the instruction of the manufacturer, an rK-39 ICT was performed using serum or plasma for which collection of venous blood is obligatory. However, our study showed excellent sensitivity and specificity levels for the rK-39 ICT test using a non-invasive procedure, i.e. urine samples. The sensitivity and specificity of urine rK-39 in our study were within the acceptance level of the serum rK-39 strip test’s sensitivity and specificity of targeted (>90%) in the different subcontinents of the world [31]. Therefore, collection of urine is comparatively easier and painless than withdrawing blood so it can help to screen *Leishmania* exposure in remote areas. Subsequently, it can contribute to the control programs for VL management and eradication [22].

In this study, the serum rK-39 test was positive for 100 patients from a total of 300 study participants. On the other hand, 107 study participants were positive for an rK-39 test by using urine. This indicates that the sensitivity and specificity of the urine-based rK-39 test were 98.0% and 95.5%, respectively. The sensitivity result of our study was supported by different findings that have been conducted in different parts of the Indian subcontinent with 96.4% [17] and 96.1% [21] sensitivity and 95% in Bangladesh [20]. Furthermore, the sensitivity of the present study corroborated the finding of the study conducted in India for the development of ELISA-based noninvasive urine for diagnosis of VL showed a sensitivity of 97.94% [22].

However, the current study showed a higher sensitivity than the previous studies conducted in Sudan with a sensitivity of 72.1% on urine [15]. The possible explanation for the difference in sensitivity of the test may be due to the difference in Leishmania species, study population, geographic areas and use of different types of rK-39 test strips.

A highly specific test can be useful for rule out patients who have certain diseases. This study showed that the overall specificity of the urine-based rK-39 test was 95.5% with a 95% CI of 91.6% - 97.9%. The specificity of an rK-39 test by using urine samples in our study was almost in agreement with a study that has been conducted in Bangladesh (93.3%) [20]. But, when we compare the current study with a study conducted in the endemic region of India, a higher overall specificity (100%) has been indicated [21, 22]. The difference could be explained by the homogeneity of *L*. *donavani* strains in India and their heterogeneity in East Africa [31]. So, the strain heterogeneity of VL causative agents in our country may result in a high rate of the discordant rK-39 test results by using serum and urine. Conversely, a lower rate (76.9%) of specificity was reported in a study that has been conducted in Sudan [15]. This might be due to variation in study subjects. In our study, a lot of diseases that may have outcomes of false positive or negative test results were excluded.

In our study, the urine rK-39 ICT showed a different level of specificity in each control group. It showed that none of the nonendemic healthy controls were positive, whereas 4.0%, and 10.0% of endemic healthy and other infectious disease controls gave positive reactions, respectively, which indicates false-positive reactions. These result of specificity in our study was higher than the study conducted in India that revealed the specificity of 66.7%, 77.08% and 62.2% for endemic healthy controls, non-endemic healthy controls and different diseases containing the patients suffering from malaria, tuberculosis, amoebic liver abscess, respectively in urine samples [17]. The difference between the Indian and Ethiopian levels of specificity could be explained by the difference in endemicity and prevalence of VL. Although it might be possible that these control individuals have an underlying asymptomatic leishmaniasis infection that demonstrated in different Indian subcontinents [32].

The reproducibility of the rK-39 strip test by using urine sample as compared with serum sample was excellent (k = 0.92, p<0.001) which is perfectly similar to the reproducibility of rK-39 strip test using urine for diagnosis of visceral leishmaniasis in an endemic region of India (k = 0.93) [21]. Moreover, it is corroborated with the reproducibility that had been conducted on an endemic area of Bangladesh with the excellent reproducibility (k = 0.88) of urine-based rK-39 test for diagnosis of VL [20].

Collection, storage and handling of urine samples are safe, non-invasive and advantageous over serum samples. The advantage of this is particularly to infants with *Leishmania* infection, from whom the collection of blood is difficult. The test is easy to perform, rapid, cheap and gives reproducible results. It is also easier in handling and does not involve much expertise or sophisticated instruments, thus suitable for outreach diagnosis and epidemiological surveys [22].

## Limitation of the study

In this study parasitological diagnosis of splenic aspirate was not performed due to procedure complexity and lack of experienced personnel to complete the procedure. Additionally, the molecular method was not used. Although this method is more sensitive than serological tests, it is not affordable for developing countries. In our study, the sensitivity may have been overestimated because of the comparison was based on rK-39 ICT result by using manufacture recommend sample.

## Conclusion

The present study showed that an excellent sensitivity and specificity of the rK-39 ICT test using urine as compared with serum samples. Further, the rK-39 test by using urine sample showed excellent agreement with an rK-39 test by using serum sample. This indicates that urine sample would be promising noninvasive, being safe, easy to collect and to perform in difficult field settings. The findings suggest that the urine based rK-39 test could be a practical and efficient point-of-care tool for the early screening and diagnosis of VL patients in rural areas, specifically where resources are limited. Finally, we would like to recommend a large-scale field evaluation of the urine based rK-39 ICT is required before using it as a diagnostic tool for VL patients in different endemic areas.

## Abbreviations

CI: Confidence Interval
ICT: Immune Chromatographic Test
VL: Visceral Leishmaniasis
WHO: World Health Organization
